# Reviewer acknowledgement 2013

**DOI:** 10.1186/1751-0759-8-2

**Published:** 2014-02-24

**Authors:** Koji Tsuboi

**Affiliations:** 1 Department of Psychosomatic Medicine, Toho University School of Medicine, Japan

## Abstract

**Contributing reviewers:**

The editors of *BioPsychoSocial Medicine* would like to thank all our reviewers who have contributed to the journal in Volume 7 (2013).

## 

Tatsuyuki Arimura

Japan

Akihiro Asakawa

Japan

Takanobu Baba

Japan

Johannes Bitzer

Switzerland

Tara Brinkman

United States of America

Hans-Christian Deter

Germany

Yukihiko Fujita

Japan

Atsushi Fukao

Japan

Shin Fukudo

Japan

Mikihiko Fukunaga

Japan

Teruhiko Higuchi

Japan

Reiko Hori

Japan

Masako Hosoi

Japan

Akio Inui

Japan

Yuko Ishizaki

Japan

Shuhei Izawa

Japan

Tsutomu Kamei

Japan

Kenji Kanbara

Japan

Hiroshi Kaneko

Japan

Michiko Kano

Japan

Toshihiko Katafuchi

Japan

Kenji Kawakita

Japan

Helen Kelsall

Australia

Takanori Kochiyama

Japan

Gen Komaki

Japan

Tetsuya Kondo

Japan

Richard Lane

United States of America

Lisa Lewis

United States of America

Joanna Mazur

Poland

Narumi Nagai

Japan

Shinichiro Nagamitsu

Japan

Motoaki Nakamura

Japan

Mutsuhiro Nakao

Japan

Tetsuro Naruo

Japan

Nobuo Nishi

Japan

Jessica Noggle

United States of America

Shinobu Nomura

Japan

Takakazu Oka

Japan

Kathleen Pike

United States of America

Ryoichi Sakuta

Japan

Tomotaka Shoji

Japan

Thomas Suslow

Germany

Shu Takakura

Japan

Takeaki Takeuchi

Japan

Hidetaka Tanaka

Japan

Masaaki Tanaka

Japan

Shirley Telles

India

Kazunari Tominaga

Japan

Mitsunao Tomioka

Japan

Akira Toyofuku

Japan

Eiji Wakamiya

Japan

Ikuro Wakayama

Japan

Blake Woodside

Canada

Jumpei Yajima

Japan

Hidenori Yamasue

Japan

Seiji Yoshida

Japan

Kazufumi Yoshihara

Japan

Kazuhiro Yoshiuchi

Japan

Stephan Zipfel

Germany

